# Innate Lymphoid Cells: Regulators of Gut Barrier Function and Immune Homeostasis

**DOI:** 10.1155/2019/2525984

**Published:** 2019-12-20

**Authors:** Hui Fan, Aiyun Wang, Yuan Wang, Ye Sun, Jing Han, Wenxing Chen, Shijun Wang, Yuanyuan Wu, Yin Lu

**Affiliations:** ^1^Jiangsu Key Laboratory for Efficacy and Safety Evaluation of Chinese Material Medica, School of Pharmacy, Nanjing University of Chinese Medicine, Nanjing 210023, China; ^2^Shandong Co-Innovation Center of TCM Formula, College of Traditional Chinese Medicine, Shandong University of Traditional Chinese Medicine, Shandong 250035, China

## Abstract

Innate lymphoid cells (ILCs), identified in the early years of this century as a new class of leukocyte family unlike the B or T lymphocytes, play a unique role bridging the innate and adaptive immune responses in mucosal immunity. Their origin, differentiation, and activation process and functions have caught global interest. Recently, accumulating evidence supports that ILCs are vital regulators for gastrointestinal mucosal homeostasis through interactions with other structural and stromal cells in gut epithelial barriers. This review will explore the functions of ILCs and other cells in maintaining gut homeostasis and feature the crosstalk between ILCs with other cells and potential pharmacotherapy targeting ILCs applicable in intestinal innate immunity.

## 1. Introduction

The gut barrier is a heterogeneous unit composed as a multilayer system and can be simplified as two components: a physical barrier surface and a deep functional barrier. The physical barrier surface prevents bacterial infiltration and adhesion and regulates paracellular diffusion to the host tissues while the deep functional barrier discriminates between pathogens and commensal microorganisms, organizing the immune tolerance and the immune response to pathogens [1]. There are many types of cells, microorganisms, mediators, and molecules constituting the gut barrier. The physical barrier then contains three major elements which are the intestinal mucosa, intestinal epithelial layer, and microbiota. The central element is the intestinal epithelial layer, which provides physical separation between the lumen and the body. The secretion of various molecules into the lumen reinforces the barrier function on the extraepithelial side, while a variety of immune cells provide additional protection below the epithelial layer. Among all the immune cells, a group of lymphocytes which are termed innate lymphoid cells (ILCs) have been studied heavily in recent years and have important roles and close communications with other cells in the epithelial barrier. In this review, we are going to focus on the interaction and crosstalk among ILCs and other cells in the gut barrier and describe how they influence the barrier function and immune homeostasis.

### 1.1. First Line of Defense: Gut Barrier Function in Intestinal Physiology

**Figure 1:**
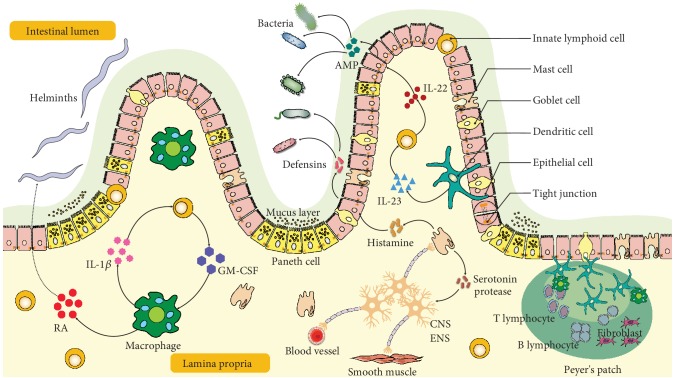
Illustration of intestinal barrier structure and functions. The intestine barrier contains the chemical barrier and the physical barrier. The chemical barrier is composed of antimicrobial peptides (AMPs) such as amphiregulin. It provides chemical agents attacking invading microorganisms including bacteria and helminths. The physical barrier includes the mucus layer and cell junctions between the epithelium. It serves as the wall spatially separating the invading microorganisms and host. There are many types of cells in the gut epithelium regulating the epithelium function. Disruption of the intestinal barrier allows the leak of gut bacteria from the lumen into the lamina propria, inducing excessive immune responses of the host immune cells. Retinoic acid (RA) released by macrophages or dendritic cells assists in host resist helminthic infection. IL-22 released by ILCs promotes epithelial cells secreting AMP in response to bacterial infection, which is regulated by IL-23 from dendritic cells. Moreover, macrophage-derived IL-1*β* promotes ILCs' production of GM-CSF, which further stimulates more macrophage differentiation from monocytes. The enteric nervous system including neuron and glial cells interacts closely with mast cells and regulates blood vessels. IL: interleukin; AMP: antimicrobial peptide; GM-CSF: granulocyte-macrophage colony stimulating factor; RA: retinoic acid; ENS: enteric nervous system; CNS: central nervous system.

The intestine represents a major gateway for potential pathogens, which also contains antigens from diets and extensive and diverse commensals that need to be tolerated. The gut barrier therefore plays important roles in intestinal physiology such as physical barrier, immune tolerance, pathogen clearance, and chronic inflammation. Its functions rely heavily on a complex group of cells and mediators in the tissue context containing structural cells such as epithelial cells, goblet cells, Paneth cells, and immune cells such as mast cells, dendritic cells, macrophages, and lymphocytes (Figure 1). We will give a brief description on the role of individual component cells in the gut barrier.

### 1.2. Intestinal Epithelial Cells

Intestinal epithelial cells constitute the majority of the cellular layer of the gut barrier. The weakening of intercellular junctions between intestinal epithelial cells will result in increased intestinal permeability and systemic exposure to bacterial antigens. The increased diffusion of bacterial components into the blood, lymph, and other extraintestinal tissues is closely related with critical illness, inflammatory bowel disease, celiac disease, food allergy, irritable bowel syndrome, and metabolic syndromes such as diabetes and obesity [2–4]. Therefore, intestinal epithelial permeability provides a novel target for disease prevention and therapy [5, 6].

In intact intestines, the intercellular junctions are primary determinants of normal barrier function. There are many kinds of intercellular junctions including the tight junction, adherens junction, gap junction, desmosome, and hemidesmosome. Tight junctions (TJs) are connected areas of the plasma membrane that stitch cells together therefore consisting a series of anastomosing strands. TJs play leading roles in paracellular permeability. Claudins, occludin, and ZO family proteins are critical components of TJs. Claudins are the most important tetratransmembrane TJs. Their extracellular domains form pores on adjacent cells and regulate TJ ion selectivity [6, 7]. Expression levels of the claudin protein are related with the intestinal barrier integrity in different ways depending on the type of claudin isoform [8]. For example, the downregulation of claudins 5 and 8 can drastically reduce the barrier integrity [9]; in contrast, claudin-2, required for the formation of paracellular water channels, is upregulated in IBDs and is highly expressed in leaky epithelial tissues and promotes inflammation [10]. Occludin is the first identified and an important protein for TJ stability. It has a dual role in the intestinal barrier. The expression of occluding was closely correlated with the barrier function both in vitro and in vivo [8, 11]. Interestingly, genetic manipulated mice which were deprived of occluding showed stability in several epithelial tissues including gastric and intestinal epithelia [12, 13]. Collectively, the functions of occludin and the mechanism by which occludin regulates the TJ are complex and still remain elusive. Unlike claudins and occludins which are integral membrane proteins and function as a gate, ZOs are peripheral membrane-associated proteins linking membrane protein to the cytoskeleton and ubiquitously expressed in epithelial and endothelial cells [14]. The various isoforms, ZO-1, ZO-2, and ZO-3, are all characterized by their ability to interact with different cellular proteins such as claudins or occludins through a multitude of protein-binding domains, such as the SH3 domain, the PDZ domain, and the leucine-zipper domain. In DSS-induced colitis mouse models, complete loss of ZO-1 expression occurred during the preinflammatory stage [15]. Adherens junctions join the actin filaments of neighboring cells together. Gap junctions are clusters of channels that form tunnels of aqueous connectivity between cells. Desmosomes are even stronger connections that join the intermediate filaments of neighboring cells. Hemidesmosomes connect intermediate filaments of a cell to the basal lamina, a combination of extracellular molecules on other cell surfaces.

### 1.3. Goblet Cells

Goblet cells secrete mucins which constitute the hydrated gel coated on the luminal surface of the intestinal mucosa. The mucus layer is the front line of innate host defense and prevents large particles and bacteria from coming into direct contact with the underlying epithelium. In the small intestine, the goblet cell-secreted Muc2 mucin, which is the first human secretory mucin to be identified and characterized, constitutes the main component of the mucus layer [16]. The mucin structure is markedly altered in colitis mouse models, and transgenic mice lacking *Muc2* gene developed colitis spontaneously [17]. Besides the secretory mucin glycoproteins (MUC2), goblet cells synthesize many bioactive molecules such as epithelial membrane-bound mucins (MUC1, MUC3, and MUC17), trefoil factor peptides (TFF), resistin-like molecule *β* (RELM*β*), and Fc-*γ* binding protein (Fcgbp) [18].

Mucin secretion is frequently coupled with increased synthesis of mucins. The biology of mucin compositions and syntheses have been summarized in details [19]. Activation of mucin synthesis can be induced Th1 cytokines (e.g., tumor necrosis factor- (TNF-) *α*) and Th2 cytokines (e.g., interleukin- (IL-) 4, IL-13), microbial products (e.g., lipopolysaccharide (LPS)), and neuropeptides. The regulation of mucin expression is controlled either by transcriptional regulation or by epigenetic regulation [18]. Due to the potent binding site in MUC2 promoters, cumulative evidences indicate that transcriptional regulation of *MUC2* is mediated by transcription factor nuclear factor- (NF-) *κ*B, a common activated transcription factor during inflammation in the gastrointestinal tract [20, 21], intestine-specific transcription factors Cdx-1 and Cdx-2 [22], forkhead box transcription factors Foxa1 and Foxa2 [23], and CREB/ATF1 [24]. Epigenetic regulation includes DNA methylation, histone modifications, and microRNA silencing. *MUC2* gene expression is regulated closely by DNA methylation and histone modifications in the 5′ flanking region of *MUC2* promoter [25]. In mucinous and nonmucinous colorectal cancer tissues, *MUC2* expression is downregulated by methylation of CpG islands in the specific regions of *MUC2* promoter [26].

### 1.4. Paneth Cells

Paneth cells reside mainly in small intestine epithelium and are located at the base of crypts of Lieberkühn (just below the intestinal stem cells in the intestinal glands) and contribute to intestinal innate immunity by secreting a diverse repertoire of antimicrobial peptides and proteins [27].

Paneth cells are vital in controlling intestinal barrier penetration by commensal and pathogenic bacteria. They sense enteric bacteria through cell-autonomous MyD88-dependent toll-like receptor (TLR) activation, triggering expression of multiple antimicrobial factors such as lysozyme and defensins (called cryptdins in mice) [28]. Defensins are the principal molecules secreted by Paneth cells. Defensins have a hydrophobic domain which can interact with phospholipids on bacterial cell membranes and thus lead to bacteria cell lysis. Paneth cells are daughter cells differentiated from intestinal stem cells [27]. Interestingly, a recent ex vivo study by Dr. Han Clevers group showed that Paneth cells lose their secretory expression signature, reenter the cell cycle, and acquire stem-like properties, contributing to the tissue regenerative response to inflammation [29].

### 1.5. Mast Cells

Mast cells in the gastrointestinal (GI) tract are located in close proximity to sensory nerve fibers, which by communicating bidirectionally play roles in the brain-gut axis [30, 31]. The interactions between mast cells and enteric neurons ensure the function of the enteric nervous system (ENS) regulation of the GI tract physiology such as motility, secretion, and microcirculation as well as immune responses [32, 33]. Moreover, the interactions are closely correlated with severity and frequency of GI tract disorders such as abdominal pain [34]. Mast cells in the GI tract comprise 1-5% of mononuclear cells in the lamina propria, submucosa, and epithelial layers [30]. Mast cells are derived from the myeloid stem cells and are similar to granulocytes. They exert their functions in two steps, which contain activation inducing degranulation and release of inflammatory mediators, including histamine, cytokines, proteoglycans, and proteases [35]. They contribute to innate and acquired immunities and are important effector cells in host defense in GI tracts overloaded every day with external stimuli such as food, pathogens, toxic substances, commensal flora, and moreover endogenous small molecules such as neurotransmitters, neuropeptides, growth factors, and hormones. Generally, mast cell activation is classically stimulated by interaction of antigens coming from allergens with its specific IgE antibody bound to the mast cell membrane through the high-affinity receptor Fc*ε*RI [36]. Besides, mast cells also express receptors for IgG (FcRI), immunoglobulin free-light chains (IgLCs), other Ig-associated receptors, complement fractions, and toll-like receptors. Activation via one of these receptors results in phosphorylation cascades and activation motifs that lead to intracellular calcium flux, activation of transcription factors such as activator protein 1 (AP-1), microphthalmia-associated transcription factor (MITF), and signal transducers and activators of transcription 5 (STAT-5) and downstream protease, cytokines, and mediator expression [30, 32]. Based on different protease contents, most mast cells can be divided into two categories: MC_T_ containing mainly tryptase and MC_TC_ containing tryptase, chymase, and carboxypeptidases [33, 37]. In the GI tract, MC_T_ comprise ~98% of all mast cells in the mucosa and ~13% of all mast cells in the submucosa [33].

### 1.6. Dendritic Cells

Dendritic cells (DCs) are key modulators that shape the immune system. In mucosal tissues, DCs play surveillance roles to sense infection and also function as the major antigen-presenting cells that stimulate the differentiation of naive T cells. They function in bridging the innate signaling and adaptive immune systems to maintain the homeostasis of the intestinal immune environment [38]. Besides, DCs are able to open tight junctions and to sample antigens directly across the epithelium both in vivo and in vitro [39]. Intestinal DC can be divided into several subsets based on the surface expression of integrins CD11c and CD103. More recently, CD24 and Sirp*α* have been introduced for better discrimination of DCs from macrophages [40].

Although DCs are located primarily in lamina propria and mucosa-associated lymphoid tissues rather than in the epithelial barrier, DCs have intimate interactions with the epithelial layer. Goblet cells were shown to transfer small soluble antigens from the intestinal lumen to CD103+ DC [41]. Chemokines secreted by enterocytes in response to TLR ligand exposure can induce the above-mentioned relocation of lamina propria DC to the epithelium [42]. Epithelial and stromal cells secrete factors, which are thought to induce DC tolerance, such as RA, TGF-*β*, PGE-2, and TSLP [43]. Establishing intestinal tolerance is critical for the prevention of intestinal diseases such as IBD, and manipulating mucosal DCs provides potential therapeutic strategies to protect against infectious diseases.

### 1.7. Macrophages

The intestine contains the largest pool of macrophages in the body. Located in the subepithelial lamina propria, intestinal macrophages are the most abundant mononuclear phagocytes. They maintain mucosal homeostasis by capturing and eliminating bacteria that cross the epithelial barrier and meet the constant phagocytosis need for epithelial renewal [43, 44]. They are important components of protective immunity and are involved in the pathology of inflammatory bowel disease (IBD) [45]. Macrophage-restricted IL-10 receptor deficiency causes severe spontaneous colitis [46]. Mouse model genetically inactivation of stat3 in macrophages will develop inflammation in the colon spontaneously and tumor lesions including invasive carcinoma with a frequency similar to that observed in human IBD patients [47].

Defining the biological roles of intestinal macrophages, characterizing the phenotype, and defining the origins of different populations of myeloid cells in the mucosa have been studied quite extensively recently [45]. Intestinal macrophages originate from yolk sacs or fetal livers at the embryonic stage and are replaced in the gut by Ly6C^+^ blood monocytes shortly after birth [48]. In adult guts, they undergo continuous renewal from monocyte-derived cells. In the process differentiation, monocytes lose Ly6C expression while other macrophage surface markers are upregulated such as MHCII, F4/80, CD11c, and CX3CR1 [43, 49].

While it has been known for many years that macrophages are present in deeper layers of the gut wall, only recently has work begun to interrogate their role in intestinal homeostasis [44]. Macrophages are also found in the submucosa, and recent depletion studies have revealed a role for these cells in maintaining the integrity of the submucosal vasculature [50].

### 1.8. Intraepithelial Lymphocytes

The intraepithelial lymphocytes (IELs) that reside between the intestinal epithelial cells (IECs) form one of the main branches of the immune system [51]. The small intestine contains approximately 1 IEL per 10 intestinal epithelial cells (IECs), and this ratio is lower in the colon [52]. IELs are resident in the intestinal epithelium and do not recirculate [53]. They express several characteristic surface receptors such as the chemokine receptor CCR9, which interacts with CCL25 produced by IECs and thus assists in recruiting IELs to the gut mucosa [52]. Intestinal IELs also express integrin *α*E*β*7 (*α*_E_ is also known as CD103), which interacts with E-cadherin on enterocytes to facilitate entry and retention in the intestinal epithelium. Approximately 90% of all IELs express T cell receptors (TCRs), and these cells have been the main focus of studies on IEL biology.

### 1.9. Neurons

Intestinal neurons can be classified as intrinsic and extrinsic. The former can also be termed as enteric neurons which have cell bodies within the gut, while the latter refers to neurons which have cell bodies located outside the intestine such as sympathetic and parasympathetic autonomic nervous systems [54]. The intestine is the largest immune cell compartment with millions of enteric neurons in the body. Therefore, it is also called the second brain [55]. Enteric neurons include myenteric and submucosal neurons. Submucosal neurons control gut secretions, nutrient absorption, and local blood flow whereas myenteric neurons orchestrate smooth muscle contractions [56, 57] (Figure 1).

Apart from enteric neurons, there are enteric glial cells found in enteric ganglia in lamina propria and smooth muscle [54]. They outnumber enteric neurons. Together, they constitute the enteric nervous system (ENS), which could continuously extend from the base of the crypts to the mucosa. Glial cells are vital to intestinal barrier integrity. Complete deletion of glial cells leads to fatal jejunoileitis in mice due to barrier integrity disruption [58, 59]. However, partial conditional depletion of enteric glial cells failed to induce inflammation and barrier disruption in intestines [60]. Enteric glial cells participate in sensing pathogens and produce neurotrophic factors and help maintain the epithelial barrier integrity. Finally, when neurons are damaged, enteric glial cells can transdifferentiate into enteric neurons to compensate [54].

### 1.10. Innate Lymphoid Cells in the Gut Barrier

Innate lymphoid cells (ILCs) are a relatively recently discovered lymphocytes compared to its other counterpart lymphocytes such as Th cells or Th17 cells. ILCs do not express the type of diversified antigen receptors expressed on T cells and B cells, and they are largely tissue-resident cells and are deeply integrated into the residential tissues [61]. While adaptive lymphocytes are most numerous in lymphoid organs—hence the derivation of the term “lymphocyte”—ILCs are relatively rare in primary and secondary lymphoid tissues. Consequently, their existence has been overlooked for many years, as immunologists focused efforts on peripheral blood and lymphoid organs. However, it is now recognized that their positioning in peripheral tissues particularly abundant at barrier surfaces in the lung, skin, and intestinal tract affords a strategic advantage for ILCs as early responders to tissue perturbation. Indeed, as a result of their location and effector phenotype, ILCs are rapid-responding cells and they produce cytokines within hours of activation, in contrast to the days required for naive adaptive lymphocytes to be primed, expand, differentiate, and enter tissues [62].  Table 1 lists some of the remarkable findings in ILC research history about ILC discovery, identification, and functions.

ILCs have been identified with many subtypes mainly divided into three groups. Group 1 ILCs include noncytotoxic ILC1s and cytotoxic conventional NK cells. Conventional NK cells were first discovered in 1975 and have been studied well with a longer history compared to other types of ILCs. Many of their functions, interaction with microbiota, antitumor responses, and involvement in the gut barrier have been investigated and reviewed in details elsewhere [74, 75]. Hence, we are not going to review this part here. We will term group 1 ILCs as ILC1s thereafter. ILC1s are regulated by T-bet and can produce IFN-*γ*, GM-CSF, granzyme, and perforin in response to IL-12, IL-18, or other activators such as pathogens or tumors. They cooperate with Th1 cells against intracellular microbes such as viruses, bacteria, or parasites [71, 73, 76, 77]. Group 2 ILCs (ILC2s), similarly to Th2, express Gata3 and can produce IL-4, IL-5, IL-13, IL-9, and amphiregulin in response to IL-25, IL-33, and TSLP (thymic stromal lymphopoietin). ILC2s are essential in the immune response against large extracellular parasites and allergens. Their production of antimicrobial peptides promotes tissue damage repair [78, 79]. A recent discovery published by Huang et al. on *Science* by using mouse models and advanced imaging techniques to monitor ILC activation and movement showed that ILC2s originate in the gut, enter lymphatic vessels, circulate in the bloodstream, and can migrate to other organs to help fight infection against helminth [80]. The trafficking of ILC2s is in a partly sphingosine 1-phosphate- (S1P-) dependent manner [80]. Group 3 ILCs (ILC3s), mirroring Th17, express ROR*γ*t, the lymphotoxins *α* and *β*, IL-17 and IL-22, GM-CSF, and TNF-*α*. They can be activated by IL-23 and IL-1*β* or by NCR ligands, and they combat extracellular microbes, such as bacteria and fungi [77] (Table 2).

The classification of ILCs into ILC1, ILC2, and ILC3 subsets is a simplified theoretical approach for understanding ILC diversity. ILC function and differentiation programs are more complicated during immune responses. Heterogeneity and plasticity of ILCs have been identified in both human and mouse studies. Tissue-resident T-bet+ ILC1s may derive from four sources in humans: ILC precursors (ILCP); converted ILC2s exposed to IL-12 and IL-1*β*; converted ILC3s exposed to IL-2, IL-15, and IL-23; and NK cells exposed to TGF-*β* [81, 82]. Bernink and colleagues reported bidirectional plasticity between ILC1 and ILC3 in the intestinal lamina propria with different environment stimuli such as IL-23, IL-1*β*, retinoic acid, or dendritic cells [83].

## 2. Crosstalk between Innate Lymphoid Cells and Other Immune Cells

### 2.1. Crosstalk between Innate Lymphoid Cells and Dendritic Cell

#### 2.1.1. ILC1s and DCs

**Figure 2:**
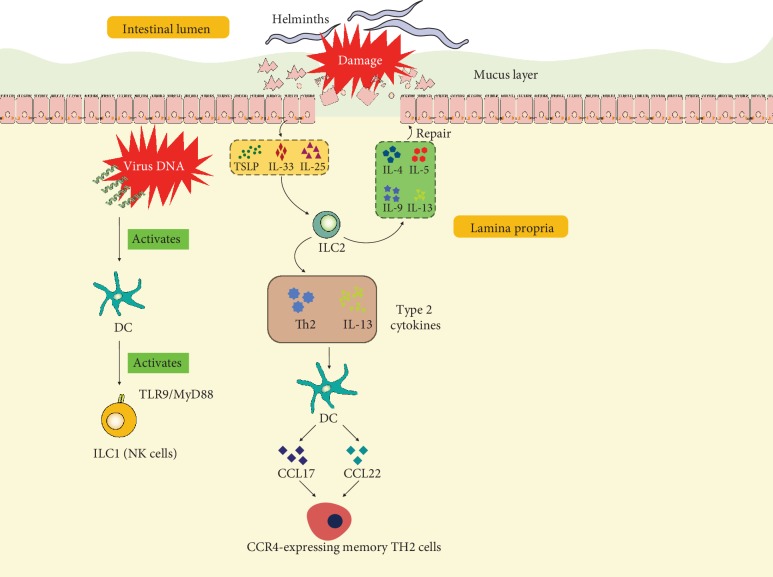
Illustration of interactions of ILCs and DCs in the intestinal tract. At the initial site of viral infection, virus DNA activates DCs, further activating ILC1 (mainly NK cells) by the TLR9/MyD88 pathway. After damage caused by helminths, epithelial cells produce TSLP, IL-33, and IL-25. These cytokines stimulate ILC2s producing type 2 cytokines including IL-4, IL-5, IL-9, and IL-13 which participate in repair of epithelial cells and mucus layer. In addition, ILC2-derived Th2 and IL-13 stimulate DCs, inducing the release of CCL17 and CCL22 and recruitment of CCR4-expressing memory TH2 cells. IL: interleukin; TSLP: thymic stromal lymphopoietin; DC: dendritic cell; ILC: innate lymphoid cell; CCL: chemokine (C-C motif) ligand; Th: helper T cell.

ILCs are characterized by prompt response after infection or injury. Tissue-resident ILC1 confer early host protection at initial sites of viral infection [84]. In a mouse model infected with pathogenic DNA viruses, Wong et al. have found that migratory dendritic cells (mDCs) induce expression of NKG2D ligands after sensing the double-stranded DNA virus via TLR9/MyD88 and promote IFN-*γ* expression in classical NK cells and group 1 ILC (mainly NK cells) already in draining lymph nodes (dLNs) through NKG2D (Figure 2). Inflammatory monocytes are also recruited to dLNs by mDCs in a TLR9/MyD88-dependent manner responding to IFN-*γ* [85].

#### 2.1.2. ILC2s and DCs

Crosstalk between ILC2s and DCs is believed to be necessary in the host to combat parasitic helminth infection executed by type 2 immune responses [78]. DCs are well-defined for antigen presentation and type 2 chemokine production during the memory Th2 cell recall-response, and it is also known that DCs can be stimulated by type 2 cytokines to produce chemokines CCL17 and CCL22, which attract its cognate-receptor CCR4-expressing memory TH2 cells. ILC2s act upstream of DCs and are essential for their production of memory TH2 cell chemoattractant CCL17. At the barrier sites, ILC2s respond to helminth infection and become activated by alarmins including IL-25, IL-33, and TSLP secreted by epithelium in the gut as an important early cellular event and produce high amounts of type 2 cytokines [86] (Figure 2). Halim et al. have reported that ILC2s-produced IL-13 has been linked to the migration of DCs in allergic asthma [87]. This interaction has been extended by Oliphant et al. that ILC2s and T cells cooperate through MHCII-dependent activation to promote DC migration to the draining lymph nodes to potentiate the Th2 generation from naïve T cells against helminth infection [78]. However, how IL-13 controls the migratory function of DCs still remains elusive.

#### 2.1.3. ILC3 and DCs

The interactions between ILC3 and DCs are discussed below in ILC3 and Macrophages.

### 2.2. Crosstalk between Innate Lymphoid Cells and Macrophage

#### 2.2.1. ILC1 and Macrophages

**Figure 3:**
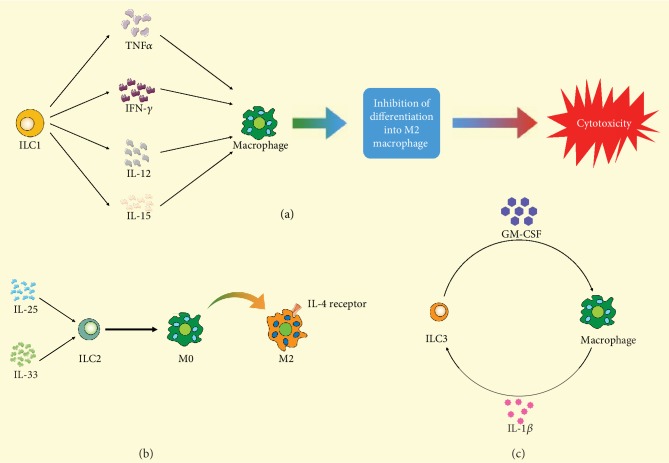
Illustration of interactions of macrophages and different ILCs. (a) TNF-*α*, IFN-*γ*, IL-12, and IL-15 released from ILC1 stimulate macrophage differentiating into cytotoxic macrophage and inhibit differentiation into M2 macrophage differentiation. (b) After being stimulated by IL-25 and IL-33, ILC2 promotes the transformation of M2 macrophage from M0 macrophage. (c) The crosstalk of ILC3 and macrophage is mainly induced by GM-CSF and IL-1*β*. ILC: innate lymphoid cell; TNF: tumor necrosis factor; IL: interleukin; M0: M0 macrophage; M2: M2 macrophage; GM-CSF: granulocyte-macrophage colony stimulating factor.

Studies on ILC1 and macrophages in intestinal tract have been scarce. Recent studies in inflammatory bowel disease animal models and intestinal infection with parasites such as *Toxoplasma gondii* have shown that ILC1s secrete IFN-*γ* and TNF-*α* and contribute to the inflammatory response and pathology in response to IL-12 and IL-15 together with macrophages [71, 88–90] (Figure 3(a)). However, studies of their interactions in obesity have shown promise. ILC1 displayed cytotoxic activity toward adipose tissue macrophages. During obesity, this killing ability was impaired and ILC1s were reported to be the major contributors for IFN-*γ* upregulation resulting in the expansion of proinflammatory M1 macrophages, and this could lead to the accumulation of pathogenic proinflammatory macrophages [91]. This interaction contributes to M1 macrophage polarization and systemic insulin resistance [92].

#### 2.2.2. ILC2s and Macrophages

ILCs can promote plastic macrophages to differentiate into alternatively activated macrophages (or M2 macrophages) in some helminth infection models to provide protective functions and tissue repair responses against helminth infection [93]. IL-25- or IL-33-activated ILC2s were found to promote M2 polarization and Treg cell expansion contributing protective immunity [94]. IL-33-activated ILC2s induced M2 polarization through IL-4 receptor signaling and directly regulated beige fat biogenesis [95]. IL2Cs has also been described to promote M2 macrophage accumulation in visceral adipose tissue during helminth infection [96, 97] (Figure 3(b)). In an airway barrier, alveolar macrophages can secrete IL-33 which will elicit direct activation of ILC2cs to produce substantial amounts of IL-13 [97]. This crosstalk in gut barriers needs to be confirmed.

#### 2.2.3. ILC3 and Macrophages

Intestinal mucosal tissue-resident macrophages together with DCs are the two main cell populations to detect microbial signals and to capture and process extracellular antigens. Meanwhile, macrophages and DCs contribute to the maintenance of immune tolerance by the induction or expansion of FoxP3^+^ Treg cells in the intestine by producing retinoic acid (RA). GM-CSF (or Csf-2) is needed to maintain DCs and macrophage numbers in the colon as well as for the Treg cells. A seminal work by Mortha et al. demonstrated that ROR*γ*t^+^ innate lymphoid cells (ILC3s) are the primary source of GM-CSF in the gut and that ILC-driven GM-CSF production was dependent on the ability of macrophages to sense microbial signals [98]. Macrophages detect microbial signals through a TLR-MyD88-dependent manner and produce interleukin-1*β*, which can act on ILC3s [98] (Figure 3(c)).

### 2.3. Crosstalk between Innate Lymphoid Cells and Epithelial Cells

#### 2.3.1. ILC1s and Epithelial Cells

ILC1s are enriched in the upper GI tract [99]. In murine models, ILC1s protect epithelial cells. *Helicobacter typhlonius* is commensal in the murine microbiota that closely resembles *Helicobacter pylori*, the frequent colonizer of the human stomach associated with gastritis, peptic ulcer, and gastric cancer. Mice lacking T-bet, the transcription factor controlling ILC1s differentiation, develop colitis triggered by *Helicobacter typhlonius* [100]. This result shows that ILC1 participate in the defense against bacterial infection. During pathological bacteria *Salmonella* infection at the intestinal tract, ILC1s are the main source of IFN-*γ*, which drives the secretion of mucus-forming glycoproteins required to protect the epithelial barrier [101].

#### 2.3.2. ILC2s and Epithelial Cells

ILC2 activation is dependent on epithelial-derived cytokines, such as IL-25, IL-33, and TSLP [97], prostaglandin D2 (PGD2) [102], and leukotriene D4 [103]. After activation, ILC2 secrete type 2 cytokines such as IL-4, IL-5, IL-9, and IL-13, which have tissue repair functions and will eventually protect epithelial cells [97].

#### 2.3.3. ILC3 and Epithelial Cells

**Figure 4:**
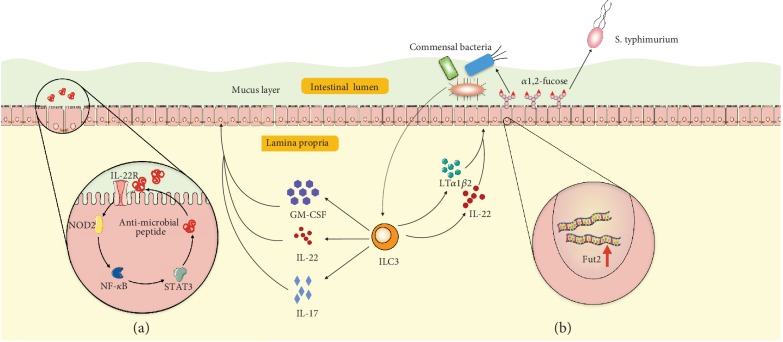
Illustration of epithelial cells and ILC3 interactions. (a) After bacterial infection, ILC3-derived GM-CSF, IL-22, and IL-17 stimulate epithelial cells by activating the IL-22R-NOD2-NF-*κ*B signaling pathway and result in epithelial cells secreting antimicrobial peptides into the mucus layer. (b) ILC3-derived IL-22 and lymphotoxin (LT*α*1*β*2) induce fucosyltransferase 2 (Fut2) gene expression in epithelial cells and result in fucose production in the intestinal tract. Fucose can be utilized by commensal bacteria but not by pathological bacteria such as *Salmonella typhimurium*. ILC: innate lymphoid cell; GM-CSF: granulocyte-macrophage colony stimulating factor; NOD2: nucleotide oligomerization domain-containing protein 2; NF-*κ*B: nuclear factor- (NF-) *κ*B; STAT3: signal transducers and activators of transcription 3.

ILC3s protect the intestinal epithelial cells and maintain the homeostasis against various pathogens. The protective role of ILC3s on epithelial cells is fulfilled by signature cytokine IL-22 released by ILC3s. Upon activation, ILC3s secrete IL-22, IL-17, and GM-CSF. IL-22 is a member of the IL-10 family and displays a homologous secondary structure, binding to its heterodimeric receptors IL-22R1 and IL-10R2 on epithelial cells. IL-22 signaling orchestrates the production of mucin and mediates epithelial cell proliferation and survival upon infection [104]. Mechanistically, IL-22 promote the production of nucleotide oligomerization domain-containing protein 2 (NOD2), which functions as a mammalian cytosolic pathogen recognition molecule. NOD2 associates with the caspase activation and recruitment domain of RIP-like interacting caspase-like apoptosis regulatory protein kinase (RICK)/RIP2 and activates nuclear factor- (NF-) *κ*B in epithelial cells [105]. The activation of NF-*κ*B induces epithelial cells to produce antimicrobial peptides and mucin. Moreover, ILC3-derived IL-22 can induce STAT3 phosphorylation and activate Lgr5^+^ intestinal stem cells for epithelial regeneration to impede tissue damage [106, 107] (Figure 4(a)). In addition, ILC3s protect epithelial cells from gut bacteria by adjusting intestinal epithelial cell glycan metabolism. ILC3s have been reported to induce the expression of fucosyltransferase 2 (*Fut2*), which catalyze fucosylation in intestinal epithelial cells in mice [108]. This induction requires the cytokines IL-22 and lymphotoxin produced by ILC3s. Fucosylation is a major mechanism of commensal bacteria utilizing dietary carbohydrate in the host. Disruption of intestinal fucosylation results in increased susceptibility to infection by pathological bacteria such as *Salmonella typhimurium* [108] (Figure 4(b)).

### 2.4. Crosstalk between Innate Lymphoid Cells and Gut-Associated Lymphoid Tissue (GALT)

GALT is a major component of the mucosa-associated lymphoid tissue (MALT) in the gut. It is the sensor for luminal content and is critical to lymphoid maturation, activation, and differentiation. It comprises isolated and aggregated lymphoid follicles, cryptopatches (CPs), and tertiary lymphoid tissue. ILCs play a central role within GALT. Prenatal GALT development is dependent on ILC lymphoid-inducer function. Postnatally, these cells rapidly respond to commensal and pathogenic intestinal bacteria, parasites, and food components by polarized cytokine production such as IL-22, IL-17, or IL-13 and further contribute to GALT formation and function [109].

### 2.5. Crosstalk between Innate Lymphoid Cells and Neurons in the Gut

At mucosal barriers, ILCs reside in close proximity to neurons and glial cells, and the crosstalk composes the functional neuron-ILC units [54, 110].

**Figure 5:**
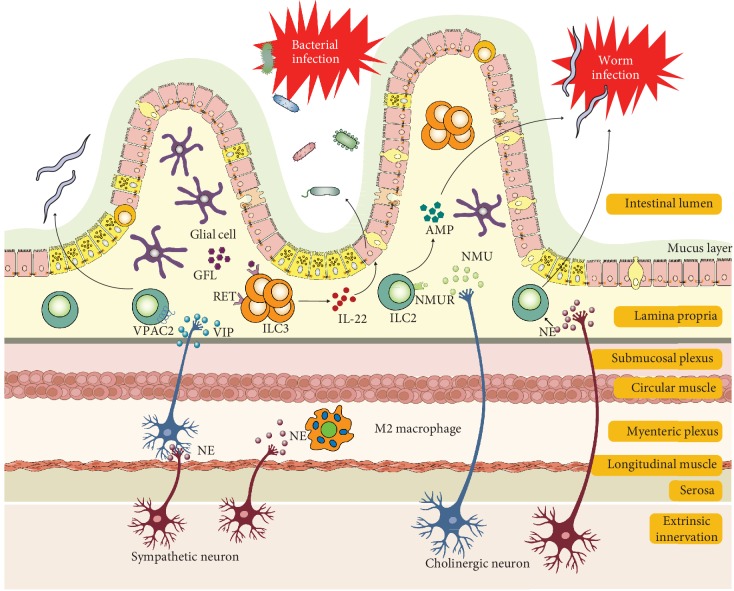
Illustration of crosstalk of ILC2 and ILC3 with neurons in helminth infection and gut inflammation, respectively. This image was modified from Reference [54]. Enteric cholinergic neuron-derived NMU activates ILC2 responses and protects against helminth infection. Lamina propria ILC2 function is also regulated by VIP and NE. Glial cell-derived neurotrophic factors stimulate IL-22 production by lamina propria ILC3s, promoting barrier integrity. SNS-derived NE induces a tissue-protective phenotype (M2) in muscularis macrophages. Abbreviations: AMP: antimicrobial peptide; NE: norepinephrine; NMU: neuromedin U; NMUR: neuromedin U receptor; SNS: sympathetic nervous system; GFL: glia cell-derived neurotrophic factor ligand; VIP: vasoactive intestinal peptide; VPAC2: vasoactive intestinal peptide receptor 2.

In response to helminthic infection, intestinal cholinergic neurons regulate ILC2 function via production of neuromedin U (NMU) [111, 112]. NMU signals through NMU receptor 1 (NMUR1) expressed in ILC2s and leads to a rapid and potent production of type 2 inflammatory cytokines, IL-5 and IL-13, and of the tissue-protective cytokine amphiregulin (Figure 5) [111, 112]. In vivo activation of this signaling axis enhances ILC2 responses and confers immediate tissue protection against helminthic infection. Subsequently, neuron-ILC2 units were identified as part of a neuron-based regulatory circuit that dampens ILC2-mediated type 2 inflammation [113]. ILC2s express the *β*2-adrenergic receptor and colocalize with adrenergic neurons in the intestine. Abrogation of *β*2-adrenergic receptor-mediated signaling resulted in increased ILC2 responses, type 2 inflammation, and lower helminth infection burden, effects that were reversed by *β*2-adrenergic receptor agonist treatment [113]. Together, these studies demonstrate that ILC2s can integrate the cholinergic and sympathetic neuronal pathways to fulfill complex regulatory functions against helminth infection.

A cutting-edge study by Ibiza et al. revealed that enteric ILC3s are part of neuroglia-ILC3 units which are orchestrated by neurotrophic factors [114]. Enteric glial cells sense microbial and host alarmin cues, which leads to increased glia-derived production of neurotrophic factors that in turn induce IL-22 production by RET (a receptor for neurotrophic factors)-expressing ILC3s. Consequently, this glia-ILC3 axis is necessary for intestinal tissue repair upon inflammatory and infection insults [114].

## 3. Regulation of Innate Lymphoid Cells and Pharmacological Potentials in Intestinal Innate Immunity

Due to the close interactions with other cells and prompt response to enteric bacteria or injury in the intestinal tract, intestinal ILCs may be targeted to manipulate immune responses early during vaccination, immunotherapy, and inflammatory pathology. Therefore, it is imperative to study comprehensively the fundamental molecular signals that regulate ILC diversity and functions. Although ILC-specific targets have not yet been identified, the activation pathways and effector molecules that can modulate ILC may provide potential therapeutic benefits.

### 3.1. ILC Transcriptional Checkpoint Targeting Strategy

Based on the transcription factors that govern the cell differentiation, function, and signature cytokine production, ROR*γ*t inhibitors have been identified primarily to block Th17-mediated inflammatory pathology [115–117]. These inhibitors can be used to block ILC3s as well, although there is a study showing that inhibition of ROR*γ*t selectively targets IL-17 producing iNKT and *γ*t-T cells but not IL-22-expressing cells [118]. Similar strategies targeting the important transcriptional checkpoint may be followed such as modulation of the activity of NK cells and ILC1s by targeting T-bet [119]. However, selective loss of T-bet in ILC1s leads to the expansion and increased activity of ILC2s [120]. The controversy or unexpected results demonstrate that we still need to study comprehensively the functions and signaling pathways that regulate the pathogenic or protective immune responses.

### 3.2. Lipid Mediators

Lipid mediators such as prostaglandin D2 (PGD2) that could regulate ILC2 responses were firstly reported in 2013 [121]. PGD_2_ activates ILC2s from human peripheral blood and increased IL-13 production in the presence of IL-33 and IL-25 [121, 122]. Arachidonic acid metabolite leukotriene D4 (LTD4) was also shown to be able to promote ILC2 activation through the cysteinyl leukotriene receptor 1 (Cys-Lt1R) [123]. Montelukast, a leukotriene receptor antagonist, binds competitively and selectively to Cys-Lt1R. Thus, montelukast may be capable of modulating ILC2 activity. Besides, the arachidonic metabolites lipoxin A4 (LXA4) and macrophage mediator resolving inflammation-1 (maresin-1 or MaR1) can impair the activation of ILC2s [121, 124]. Therefore, a variety of lipid mediators or inhibitors of these mediators may be developed as ILC modulators [122].

### 3.3. Cytokines

The cytokines inducing the development and activity of specific subsets of ILCs may also be targeted—such as IL-12 and IL-15 for ILC1s; IL-25, IL-33, and TSLP for ILC2s; and IL-1*β* and IL-23 for ILC3s, respectively. Interestingly, IL-2, not a classical inducer of ILC activation, was shown to be a critical regulator of ILC2 during pulmonary inflammation [125]. Although the precise involvement of ILCs in specific diseases still remains elusive, treatment blocking these pathways showed some effects in different scenarios besides the intestinal tract. Treatment of multiple sclerosis patients with daclizumab, an antibody targeting IL-2R*α* (CD25), resulted in an increase in the numbers of NK cells that correlated with drug efficacy [126]. Blockade of CD25 inhibits effector T cell activation, regulatory T cell expansion and survival, and activation-induced T-cell apoptosis. Because CD25 blockade reduces IL-2 consumption by effector T cells, it increases IL-2 bioavailability allowing for greater interaction with the intermediate-affinity IL-2R and therefore drives the expansion of CD56^bright^ NK cells. Unfortunately, daclizumab was withdrawn from the market in 2018 due to severe secondary autoimmune disease directed against the central nervous system [127]. In addition, antibodies against IL-25 and IL-33 have shown efficacy in mouse models of allergic lung inflammation [128, 129], and antibody to TSLP intravenously given before allergen challenge in mild asthmatic patients improves asthma symptoms [130].

Apart from cytokines inducing ILC development, effector cytokines such as IFN-*γ*, IL-5, and IL-13, or IL-17, produced by ILCs may also be targeted. For example, mepolizumab (antibody to IL-5, NCT01000506) and lebrikizumab (antibody to IL-13, NCT02104674) have been shown effective in clinical trials against asthma [131, 132].

### 3.4. Microbial Compounds

The soluble excretory/secretory products of the helminth parasites impair the activity of ILC2s in response to airway challenges by suppression of IL-33 production [133]. Alternatively, microbial compounds may be used to boost one type of ILC in order to block the other types of ILCs.

## 4. Conclusion

In the past decade, accumulating studies have been carried out to delineate the biology of ILC differentiation, function, and regulation. Yet, still much remains to be investigated. Many discoveries are based on mouse models, and more needs to be described in human scenarios. The prompt response characteristics and antigen-independent activation place ILCs upstream of adaptive response. ILCs possess only few sensory elements for the recognition of nonself, and therefore, ILCs depend on extrinsic cellular sensory elements residing within the tissue [134]. Their crosstalk with T cells, DCs, and other cells need to be deciphered further. ILCs contribute significantly to human health and disease. They play protective roles in some mucosal infections while playing detrimental roles in IBD. Development of modulators to block the detrimental roles of ILCs is of great clinical benefit.

## Figures and Tables

**Table 1:** List of some important studies in ILC research history.

Years	Events	Reference #
1975	Discovery of NK cells as the first subsets of ILCs	[63]
1997	Discovery of LTi cells (later defined as one subset of ILC3s) which are essential for the development of lymph nodes during embryogenesis	[64]
2006	Characterization of GATA3 and CD127 on ILC2s	[65]
2009	ILC3s are the source of endogenous IL-22 and constrain inflammation at the mucosal site	[66]
2010	Identification of ILC2s in mice that produce type 2 cytokines and contribute to antihelminth immunity and type 2 inflammation	[67]
2010	Identification of a role for ILC3-like cells in promoting intestinal inflammation	[68]
2011	First description of a tissue-protective role for ILC2s, describing how ILC2s produce amphiregulin, a ligand of EGFR, and contribute to lung-tissue repair following influenza A virus infection in mice	[69]
2013	First evidence of non-NK cell ILC1s in humans and the transcription factor T-bet responsible for ILC1s differentiation	[70, 71]
2013	First evidence that ILC3s directly regulate adaptive immune responses	[72]
2013	Experts described consensus nomenclature for ILC subsets	[73]

**Table 2:** Innate lymphoid cells in the gut.

Types	Surface marker		Stimulus	Regulatory transcription factor	Signature released cytokines	Functions in the gut barrier
	Mouse	Human				
ILC1	CD160	CD103	IL-12, IL-15	T-bet	IFN-*γ*, TNF-*α*, GM-CSF, granzyme, perforin	Defense against virus, pathogens
	NKp46	CD160				
	NK1.1	CD56				
		NKp46				
		NKp44				

ILC2	IL17RB	IL17RB	IL-25, IL-33, TSLP	GATA3	IL-4, IL-5, IL-13, IL-9, amphiregulin	Helminth expulsion
	IL-33R	IL-33R				
	CD25	CD25				
	CD127	CD127				
		CRTH2				

ILC3	NKp46	NKp44	IL-1*β*, IL-23, NCR ligand	ROR*γ*T	IL-17, IL-22, GM-CSF, TNF-*α*	Defense against bacteria, fungi

## Abbreviations

ILCs: Innate lymphoid cells
TJs: Tight junctions
MUC2: Mucin glycoproteins
TFF: Trefoil factor peptides
RELM*β*: Resistin-like molecule *β*
Fcgbp: Fc-*γ* binding protein
TNF-*α*: Tumor necrosis factor-*α*
LPS: Lipopolysaccharide
NF-*κ*B: Nuclear factor-*κ*B
TLR: Toll-like receptor
DCs: Dendritic cells
IBD: Inflammatory bowel disease
IELs: Intraepithelial lymphocytes
IECs: Intestinal epithelial cells
TCRs: T cell receptors
TSLP: Thymic stromal lymphopoietin
S1P: Sphingosine 1-phosphate
mDCs: Migratory dendritic cells
dLNs: Draining lymph nodes
RA: Retinoic acid
PGD2: Prostaglandin D2
NOD2: Nucleotide oligomerization domain-containing protein 2
RICK: Regulatory protein kinase
Fut2: Fucosyltransferase 2
LTD4: Leukotriene D4
Cys-Lt1R: Cysteinyl leukotriene receptor 1
LXA4: Lipoxin A4
Maresin-1 or MaR1: Macrophage mediator resolving inflammation-1.
